# Does caregivers’ mental health impact the quality of life of adolescent and young adult cancer survivors? Preliminary results of a dyadic study

**DOI:** 10.1192/j.eurpsy.2025.746

**Published:** 2025-08-26

**Authors:** M. C. Neves, S. Monteiro, F. Nunes, B. Macedo, E. Rodrigues, M. C. Rodrigues, S. Moutinho, A. P. Fernandes, M. D. J. Moura, A. Carneiro, P. Medeiros, M. Jerónimo, S. Santos, J. B. Prins, C. M. D. Sales

**Affiliations:** 1 Center for Psychology, University of Porto, Faculty of Psychology and Education Sciences of the University of Porto, Porto; 2 CINTESIS@RISE, Department of Education and Psychology, University of Aveiro, Aveiro; 3 Center for Global Studies, Department of Social Sciences and Management, Open University, Lisboa; 4 Serviço de Onco-Hematologia, Instituto Português de Oncologia do Porto Francisco Gentil E.P.E.; 5 Serviço de Endocrinologia, HSJ - Hospital de Sao Joao Centro Hospitalar Universitario, EPE; 6 Serviço de Pediatria; 7 Serviço de Psico-Oncologia, Instituto Português de Oncologia do Porto Francisco Gentil E.P.E.; 8 Serviço de Oncologia Pediátrica, HSJ - Hospital de Sao Joao Centro Hospitalar Universitario, EPE; 9 Serviço de Psico-Oncologia, Instituto Português de Oncologia de Lisboa E.P.E.; 10 Serviço de Hematologia Clínica, HSJ - Hospital de Sao Joao Centro Hospitalar Universitario, EPE; 11 Hospital Pediátrico de Coimbra, Centro Hospitalar e Universitário de Coimbra, E.P.E., Coimbra; 12 Serviço de Endocrinologia, Instituto Português de Oncologia do Porto Francisco Gentil E.P.E., Porto, Portugal; 13 Department of Medical Psychology, Radboud University Medical Centre, Nijmegen, Netherlands

## Abstract

**Introduction:**

When an adolescent or young adult is diagnosed with cancer, they’re frequently accompanied by their caregiver. Literature shows that caregivers of adolescents and young adult cancer survivors (AYACS) frequently experience high anxiety and depressive symptoms. Being these caregivers an important source of support to AYACS during this challenging journey, one question emerges: does caregivers’ mental health impact AYACS’ quality of life (QoL)?

**Objectives:**

Considering this, this study examined the associations between caregivers’ mental health and AYACS’ QoL.

**Methods:**

Forty-eight dyads were recruited in four hospitals and one association in Portugal. AYACS were mostly women (62.5%) and off-treatment (62.5%). They were, on average, diagnosed at 18.9 years (range: 15-25) and currently 21.98 (range: 15-38). Their caregivers were mostly women (77.1%) and, on average, 47.02 years (range: 19-76). Parent-child dyads were the most frequent. The Quality of Life Questionnaire Core-30 assessed the AYACS’ QoL. Caregivers’ mental health, the Hospital Anxiety and Depression Scale, and the FCR7 scale assessed caregivers’ anxiety and depressive symptoms and fear of cancer recurrence, respectively.

**Results:**

Preliminary results show that among AYACS’ QoL, only low social functioning was significantly related to high anxiety and depression in caregivers. Treatment status was also significantly positively related to AYAs’ social functioning and negatively to caregivers’ anxiety and depression. AYAs and caregivers’ ages at recruitment were also negatively correlated with caregivers’ anxiety. Age of AYACS at diagnosis, living with caregiver, and type of caregiver were not related to AYACS social functioning nor caregiver anxiety and depression. A model was tested, showing that caregivers’ anxiety and depression predict AYAs’ social functioning, having treatment status as covariable.

**Conclusions:**

Caregivers’ mental health and treatment status were shown to be important for AYACS’ QoL, especially social functioning. This supports the need to assess how caregivers are adapting to this new stage of life and provide specialized support when needed. This could indirectly have a positive impact on the QoL of AYACS. It’s important that the support provided considers the diverse challenges these caregivers face, which can differ from other caregiver groups.

**Disclosure of Interest:**

None Declared

